# Sensitive Detection and Identification Method of Erythrocyte-like Cells upon Doxorubicin Induced Differentiation with Vibrational Techniques

**DOI:** 10.1021/acs.analchem.5c02465

**Published:** 2025-07-29

**Authors:** Adriana Adamczyk, William Tipping, Olga Mazuryk, Duncan Graham, Malgorzata Baranska, Katarzyna Majzner

**Affiliations:** † Jagiellonian University, Faculty of Chemistry, Gronostajowa 2, Krakow 30-387, Poland; ‡ Department of Pure and Applied Chemistry, Technology and Innovation Centre, 3527 University of Strathclyde, Glasgow G1 1RD, U.K.

## Abstract

Altered differentiation of blood cell precursors and their clonal expansion occurs in various types of leukemia. One treatment strategy is to induce differentiation into the mature form, which is capable of undergoing apoptosis. It has been found that low doses of doxorubicin (DOX) induce phenotypic and morphological changes in malignant erythroid precursors to erythrocyte-like cells. These are usually studied by time-consuming examination of hemoglobinisation by benzidine staining or glycophorin A expression by Western blot or flow cytometry. As an alternative, we propose an in-depth investigation of DOX-induced erythroid differentiation using Raman spectroscopy and stimulated Raman scattering microscopy, and fast and sensitive classification using the probe for mitochondrial imaging (MitoBADY). Machine learning methods, including Orthogonal Partial Least Squares, Principal Component Analysis, and Multivariate Curve Resolution-Alternating Least Squares, etc., were implemented to extract spectroscopic markers of differentiation from both single spectra and the hyperspectral images.

## Graphical abstract

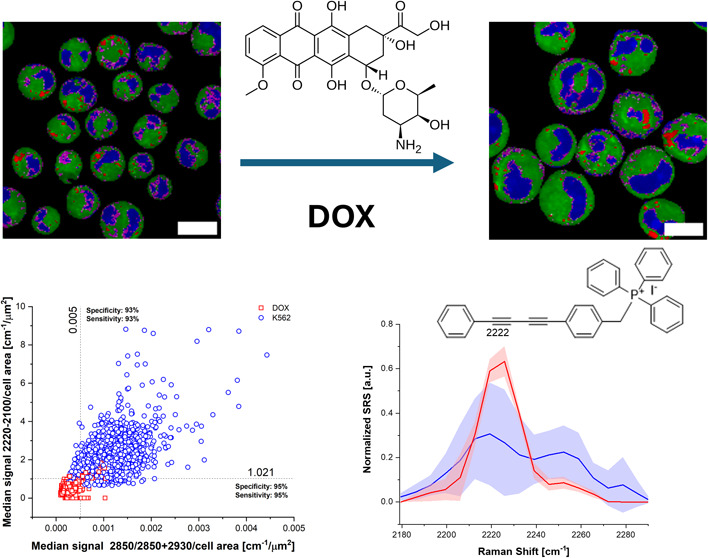

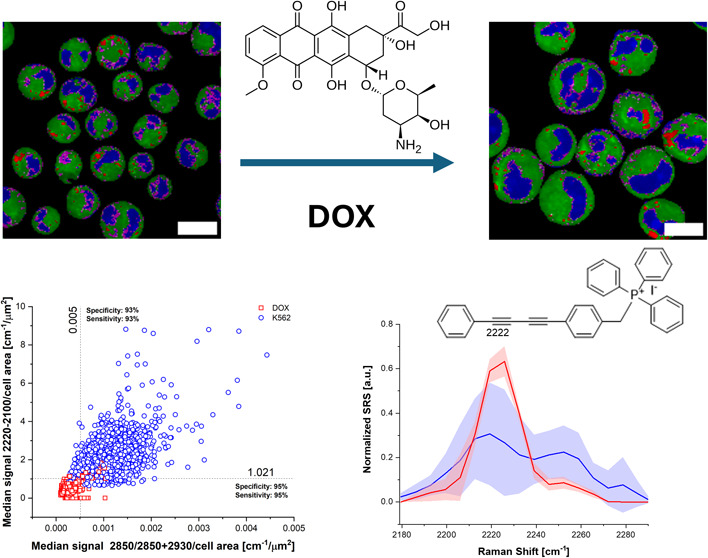

## Introduction

Erythropoiesis, a critical branch of hematopoiesis, governs the production of erythrocytes through a tightly regulated differentiation process. Understanding the molecular changes associated with erythropoiesis is essential for identifying biomarkers of differentiation and pathological conditions such as myeloproliferative disorders.^1^ To model erythroid differentiation *in vitro*, various cell lines have been developed, including K562, a human erythroleukemia cell line harboring the *Philadelphia* chromosome (*BCR-ABL* gene rearrangement) in its karyotype. While K562 cells do not fully mimic normal erythroid precursors, they provide a valuable model for studying chemically induced differentiation into erythrocyte-like cells with e.g., hemin,^2^ doxorubicin,^3^ imatinib,^3^ and cytarabine.^4^ The terminal erythroid differentiation of K562 cells can be determined on the basis of the hemoglobinization of the cells, mainly in the benzidine test (visual inspection of blue crystals)^3^ or the expression of miRNA globin and glycophorin A (CD235a).^3^ The changes associated with metabolic changes at the organelle level are also crucial. Well-functioning erythrocytes do not contain a nucleus and switch their metabolism from glycolytic to oxidative.^5^

Despite progress in understanding erythropoiesis, current diagnostic workflows for assessing erythroid differentiation remain limited by their reliance on labor-intensive, end point-based techniques. Commonly used methods, such as benzidine staining to detect hemoglobinization or flow cytometry for surface markers like glycophorin A, are time-consuming, require multiple processing steps, and often depend on cell lysis or antibody labeling. These approaches lack the capacity for real-time monitoring of biochemical and metabolic changes that accompany differentiation, particularly at the organelle level. Furthermore, they provide limited insight into mitochondrial function or intracellular metabolic remodelling, both of which are critical for understanding the dynamics of erythroid maturation and evaluating the efficacy of differentiation-inducing treatments. There is, therefore, a clear need for rapid, nondestructive, label-free technologies capable of providing both molecular specificity and spatial resolution to track differentiation processes in live cells. Vibrational spectroscopy, particularly Raman (RS) and stimulated Raman scattering (SRS) imaging, offers such capabilities by enabling the detection of biochemical markers—including hemoglobin, lipids, and mitochondrial metabolites—without the need for staining or fixation. In this study, we aim to address these diagnostic limitations by developing a spectroscopic strategy that integrates RS, SRS microscopy, and a mitochondrial membrane potential-sensitive Raman probe (MitoBADY, MB) to sensitively and specifically monitor erythroid differentiation in live K562 cells. RS has been extensively applied to mature erythrocytes^6^ due to the strong resonance enhancement of hemoglobin but remains underexplored in tracking erythroid differentiation. RS has been successfully applied to assess cellular physiology,^7,8^ characterize leukemia molecular subtypes,^9,10^ and monitor chemotherapy response.^11,12^ While neutrophil differentiation^13,14^ has been investigated with RS, applications in erythroid differentiation remain limited. To date, studies using RS to assess erythroid differentiation have either focused on mature erythrocytes or examined AML-related erythroblasts without tracking the dynamic process of erythroid maturation.^15,16^ Alattar et al. studied early erythroid differentiation on hematopoietic progenitor cells isolated from blood using gold nanoparticles, which allowed the collection of a surface-enhanced Raman scattering signal (SERS).^15^ They selected proliferating, differentiating, and mature cell populations with distinct spectroscopic profiles, primarily related to DNA, RNA, and protein. While Alattar et al. demonstrated the use of SERS on hematopoietic cells, their methodology did not address hemoglobin synthesis or mitochondrial remodelling, which are critical features of erythroid differentiation. On the other hand, Vanna et al. showed that acute myeloid leukemia (AML) erythroblasts have strong Raman signatures of hemoglobin, which they used to classify AML cells of different myeloid populations.^16^ Except that the leukemic erythroblasts were studied with RS in comparison with leukemic blasts from other cell populations^16^ or K562-based models of resistance to Imatinib^17^ or Adriamycin^18^ were compared. Mature erythrocytes have been extensively investigated using RS due to the absorption of hemoglobin in the visible region, which gives rise to the resonance Raman (RR) effect reflected in the enhancement of the bands of the absorbing component.^6^ Hemoglobin contains a highly symmetric chromophore haem prosthetic group, which provides a signal enhancement, especially when it is in resonance with the intense electronic transitions centered at about 400 nm (Soret or β band), 525 nm (Q_v_ or a band), and 575 nm (Q_0_ or b band).^6^ Another way of Raman signal enhancement is the application of nonlinear techniques such as SRS. The pump and probe lasers lead to a simultaneous in-mode vibration of all the molecules in the sample under investigation. Typically, single Raman shifts are studied simultaneously, and laser tuning is required to probe other Raman modes. Detection in the higher wavenumber range, rather than in the fingerprint region, is also more accessible.^19^

The development of lasers, Raman spectrometers, and nonlinear optics has also influenced the development of the labeling approach to probe relevant information in the silent region of the cell. Raman probes usually contain chemical groups such as alkynes, nitriles, or isotopically substituted atoms attached to biologically relevant compounds (phospholipids, cholesterol, proteins, and amino acids) to be incorporated into cell structures or chemical structures with high affinity to cellular compartments.^20–25^ Raman reports tracking intracellular pH and mitochondrial membrane potential has proven to be useful in detecting critical biochemical changes in a number of processes. So far, in a model of HL-60 all*-trans* retinoic acid (ATRA)-mediated differentiation, the MB Raman mitochondrial membrane potential probe has been used to classify myeloid precursors and neutrophil-like cells.^14^ We investigate the alternative differentiation pathways to verify variations in probe accumulation. This has the potential to serve as a differentiation indicator that is beneficial for clinical applications.

Differentiation is a dynamic process involving organelle-level biochemical changes, the understanding of which is crucial for developing new analytical strategies in leukemia research. Here, we investigate the potential of RS combined with advanced spectral analysis to monitor erythroid differentiation in K562 cells treated with low-dose doxorubicin (DOX). We employed Raman-based techniques, including spontaneous RS for detailed biochemical profiling and SRS for rapid high-throughput imaging. By leveraging high-resolution Raman imaging across the visible to infrared range, we aim to establish spectral markers of erythroid differentiation and demonstrate the feasibility of Raman-based approaches for real-time chemotherapy monitoring. To extract biochemical signatures of erythroid differentiation from Raman spectra, we employed multivariate statistical and machine-learning approaches. These included Principal Component Analysis (PCA) for dimensionality reduction, orthogonal partial least-squares-discriminant analysis (OPLS-DA) for supervised classification, and multivariate resolution-alternating least-squares (MCR-ALS) for spectral decomposition, facilitating the identification of differentiation-associated spectral features.

The unique capabilities of RS allowed us to identify differentiation markers, including hemoglobin accumulation, which was used to classify differentiated cells. Furthermore, we developed a rapid method for classifying and monitoring erythroid precursor differentiation into erythrocyte-like cells, based on lipid-to-protein ratios, cell morphology, and MB accumulation. These findings demonstrate the feasibility of translating Raman-based techniques to high-speed SRS microscopy, paving the way for future applications in clinical diagnostics and chemotherapy monitoring. Our study extends beyond previous work by integrating mitochondrial membrane potential probing with label-free vibrational profiling, specifically focusing on DOX-induced erythroid maturation in K562 cells—a model not previously explored using this combination of tools.

## Results

Erythroid precursors (K562) treated with 100 nM of DOX underwent a number of biochemical changes compared to untreated cells, including blocked proliferation, increased cell size percentage of benzidine-positive cells, and elevated expression of CD235a surface protein. (SI Doxorubicin induced differentiation, Figures S1–S2).

The validated model was investigated with single-cell RS imaging (532 nm excitation laser) to identify biochemical changes associated with erythroid differentiation. A significant increase in spectral intensities corresponding to phenylalanine (1006 and 1040 cm^–1^) and glycogen (480, 573, 850 cm^–1^) was observed following DOX treatment, suggesting metabolic and structural remodelling during differentiation. Interestingly, while DOX treatment significantly altered biochemical markers, drug bands (e.g., 440, 465 cm^–1^)^26^ were not observed. Additionally, resonantly enhanced cytochrome c (753, 1130, 1310, 1585 cm^–1^) and hemoglobin in oxidized and deoxidized forms (753, 1130, 1310, 1340, 1377, 1400, 1555, 1585, 1610 cm^–1^) (Figure 1A and Table S2) dominate in erythrocyte-like cells.

**Figure 1:**
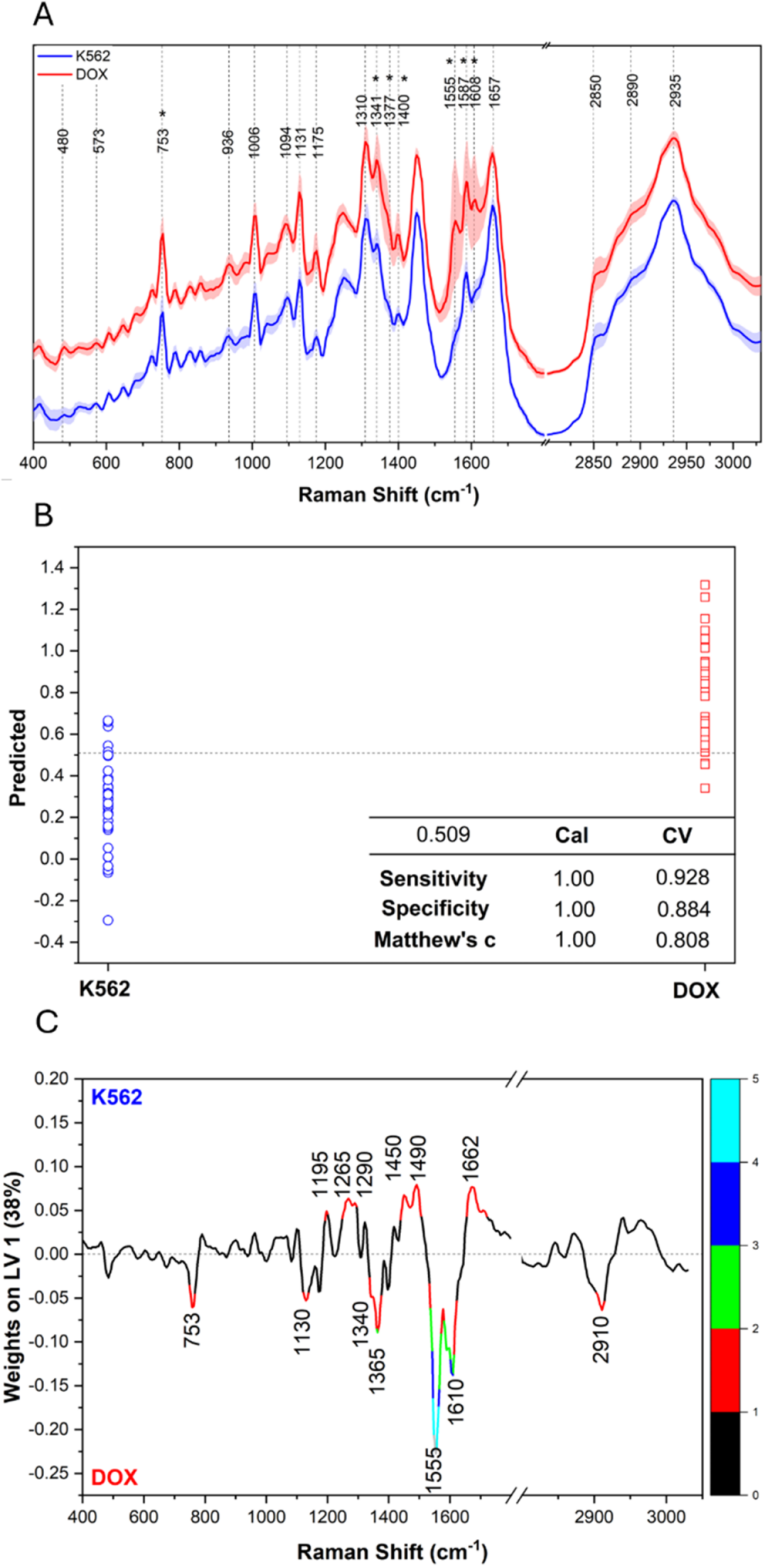
Average Raman spectrum with SD of erythroid precursors (K562, blue) and erythrocyte-like cells (DOX, red), the asterisk depicts Hb-related bands (A). Predicted values of classification for cross-validation in the OPLS-DA model (B). The classification threshold is marked with a dotted line. The model performance parameters, sensitivity, specificity, and MCC, are presented in the table. Weights on LV1 are colored according to the VIP values. VIP > 1 are assigned (C).

The 532 nm incident radiation is close to the vibronic Q_v_ band of hemoglobin in the visible spectrum, leading to a RR effect and band enhancement, but also to a fluorescence background that can alter data interpretation.^6^ We developed an OPLS-DA model to differentiate erythrocyte-like and precursor cells based on Raman spectral changes. The model, built using 4 latent variables (LVs), explained 93% of the total variance in the data set (Figure S3) and achieved high specificity (0.884) and sensitivity (0.928) in cross-validation. The Matthews correlation coefficient (MCC = 0.808) is a robust metric, particularly for imbalanced data sets,^10^ further supporting model performance (Figure 1B). The permutation test ensured that the model is not overfitted. Details on the model's robustness are available in the SI, Figure S3.

In OPLS-DA, data variation is separated into a predictive part correlated to Y response, represented on LV1, and an orthogonal part that is uncorrelated to Y.^14^ Furthermore, variable importance in projection (VIP) values greater than 1 highlight Raman features vital for classification. Erythrocyte-like cells showed VIP > 1 for hemoglobin-related bands (753, 1130, 1341, 1365, 1555, 1610 cm^–1^), confirming hemoglobin accumulation. Additionally, the 2910 cm^–1^ band of glycogen was noted on VIP as important. Erythroid precursors had VIP > 1 for protein (1265, 1450, and 1662 cm^–1^) and DNA bands (1195, 1290, 1490 cm^–1^), indicating a more nuclear and cytoskeletal composition before full maturation (Figure 1C).

The prominence of the DNA bands in erythroid precursors suggests that these cells still contain nuclear material, which aligns with their predifferentiation state. The cytoskeletal network is actively remodelling in erythroid precursors to prepare for enucleation and terminal differentiation.^27^ These bands may indicate an active phase of cytoskeletal assembly preceding maturation.

To gain deeper insights into the biochemical composition of erythroid precursors and erythrocyte-like cells, we applied MCR-ALS to hyperspectral (hs) Raman images using a contribution contrast constraint.^28^ The analysis revealed 10 distinct components (Comps.), each corresponding to characteristic cellular structures or biochemical features. We identified: (Comp. 1) nuclear structures, (Comp. 2) lipids, (Comp. 3) cell peripheral area including membrane, (Comp. 4) cell peripheral plasm including cytoplasmic proteins, (Comp. 5) hemoglobin, (Comp. 6) cytochrome c, (Comp. 7) mitochondrial-endoplasmic reticulum area, (Comp. 8) perinuclear area, and (Comp. 9) glycogen. Moreover, the component distribution images are in agreement with the fluorescent staining images of, for example, the nucleus and mitochondria (Figure S7), underscoring the feasibility of the results. Below, we describe the spectral features and spatial distribution of these components in detail (Figure 2A, B).

**Figure 2:**
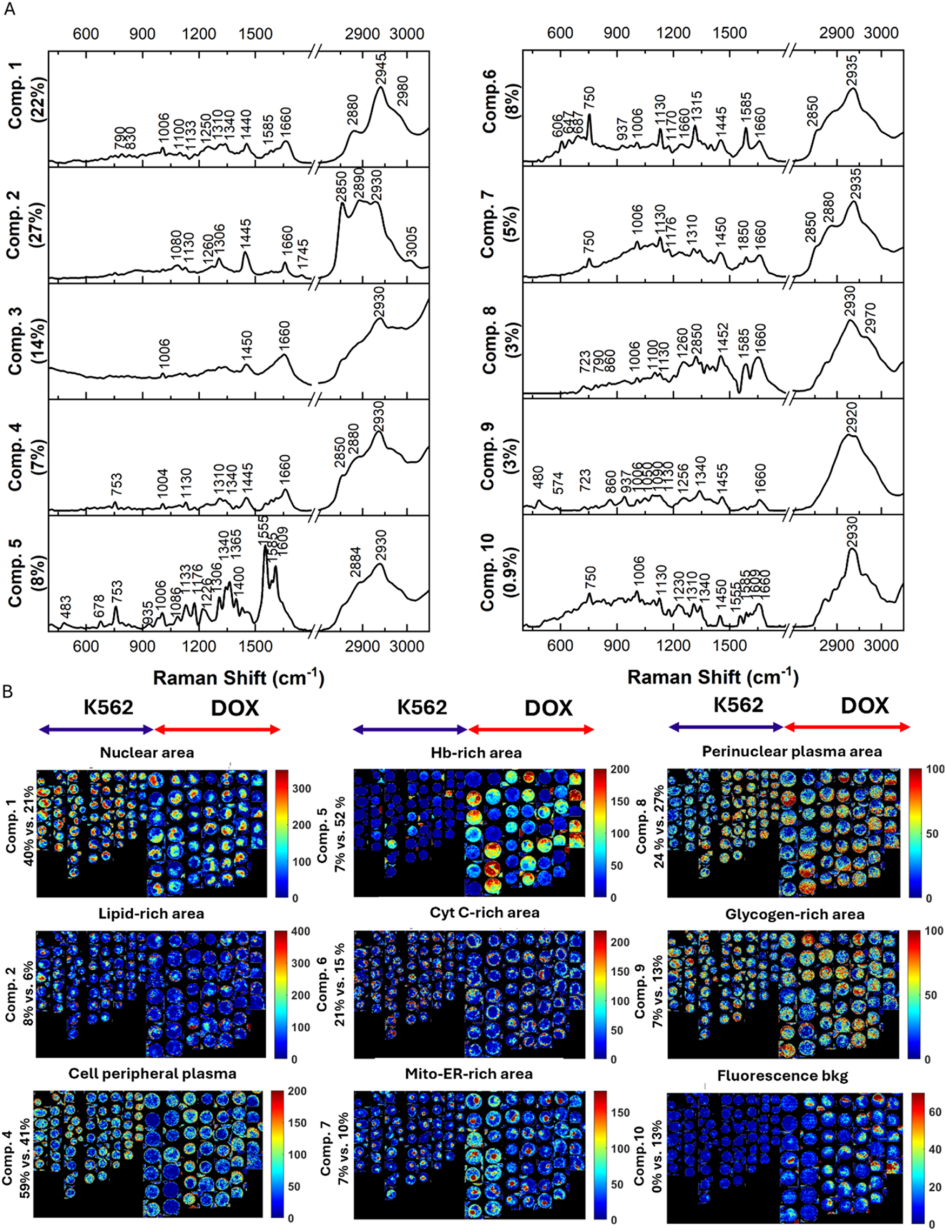
MCR-ALS of hs Raman images. Spectral components with the percentages describing the sum squared signal with respect to the total signal in the data (A) and their distribution color-coded by component concentration (B). The percentages on the left-hand side show the percentage of high-intensity pixels for each component of K562 control vs. DOX-treated. Raman images of cells from 3 independent biological replicates were pooled for the analysis.

Comp. 3, identified by the weak intensity of CH stretching bands in the 2800–3030 cm^–1^ range, provides minimal insights and is therefore not discussed further here. Comp. 1 showed features of the nuclear area that consist of DNA, RNA, and nucleoproteins (789, 1098, 1340, 1380, 1585, 2876, 2940, and 2980 cm^–1^). The spatial distribution of this component revealed a shift from a high-intensity, compact nuclear region in erythroid precursors to a more dispersed pattern in DOX-treated cells, indicative of chromatin remodelling. Histograms showed a decrease in DNA-rich pixels (threshold values in Table S3) from 40% in erythroid precursors to 21% in erythrocyte-like cells, consistent with nuclear size expansion and potential chromatin decondensation. This aligns with previous findings that DOX-induced G2/M arrest alters nuclear architecture, promoting a more relaxed chromatin state.^29^ Hemoglobin production is also linked to increased activities of the nucleolus, which is responsible for rRNA production and ribosome assembly necessary for protein production. Comp. 2 exhibited spectral features of fatty acid esters (1740 cm^–1^) and choline-containing phospholipids and/or their esters (725, 1075 cm^–1^), with higher intensity of bands characteristic to saturated lipids (1306, 1440 cm^–1^) compared to unsaturated with C=C vibrations (1260, 1660, 3009 cm^–1^). This suggests a predominance of saturated over unsaturated lipids. The noted reduction in lipid-rich pixels (from 8 to 6%) is slight, indicating that additional quantitative analysis is necessary to determine if this change is functionally significant in erythroid context differentiation. Comp. 4 was identified in the cell peripheral plasma, containing cytochrome c and hemoglobin features.

Both hemoglobin and cytochrome c are resonantly enhanced haem proteins excited at 532 nm. This leads to spectral overlap at key bands (753, 1130, 1310, 1585 cm^–1^), challenging their signal separation. However, MCR-ALS successfully separated these components, identifying hemoglobin-rich (Comp. 5) and cytochrome c-rich (Comp. 6) regions. Hemoglobin was dominant in erythrocyte-like cells (753, 1340, 1370, 1400, 1460, 1555, 1585, 1610, and 1640 cm^–1^). The proportion of pixels with high hemoglobin content increased significantly from 7% in erythroid precursors to 52% in erythrocyte-like cells (Table S2). Conversely, cytochrome c-rich pixels decreased from 21 to 15%, consistent with a shift away from mitochondrial oxidative metabolism in mature erythrocytes. The most intense cytochrome-related bands were 753, 1130, 1315, and 1585 cm^–1^, with additional peaks at 611, 647, and 1175 cm^–1^(pyrrole ring vibrations) and protein-associated bands (amide I at 1653 cm^–1^, CH_2_ deformation at 1450 cm^–1^). The silent region between 1800 and 2800 cm^–1^ in cytochrome and hemoglobin components is slightly elevated (this region is not displayed).

Interestingly, cytochrome c-rich pixels were concentrated in perinuclear regions, particularly around Comp. 7, which also contained phospholipid and broad Amide I/III bands. This suggests a high protein content in these regions, potentially related to mitochondrial-endoplasmic reticulum (Mito-ER) interactions. The broad amide I and III bands indicate a higher protein content in this region. However, further validation (e.g., colocalization studies) is needed to confirm its relation to mitochondrial-associated membranes (MAMs).^30^

Comp. 8 primarily consisted of phospholipids and amide I/III bands representing the perinuclear area with a cytoplasmic distribution. The spectral profile of Comp. 9 showed distinct Raman features of glycogen (483, 579, 856, 941, 1051, 1084, 1130, 1337, 1387, 1460 cm^–1^), with a higher cytoplasmic distribution. The percentage of pixels is higher than that of pixels in erythroid precursor cells (7% vs. 13%, Table S3). Lastly, Comp. 10. Besides the exhibited haem-related signal, we also observed significant distortions in the background profile of the cell silent region (1800–2800 cm^–1^), which were more pronounced in DOX-treated cells, and may be related to DOX fluorescence.

To minimize spectral interferences associated with RR enhancement of haem and DOX, we supplemented our analysis with line measurements using a 785 nm laser. PCA of the Raman spectra revealed separation of data along PC 1 and PC 4 (explaining 52.31 and 1.62% of the variance in data, respectively). The presence of the hemoglobin (1550 cm^–1^), predominance of the saturated lipids (1080, 1126, 1306, 1448 cm^–1^), and glycogen (480, 573, 854, 937, 1050 cm^–1^) was confirmed in the DOX cells. Spectra of erythrocyte precursors were dominated by the higher contribution of DNA-related bands (795, 1180, 1137, 1585 cm^–1^) and Phe (1000 cm^–1^) (Figure S4A–D). The DOX-specific bands (Figure S5) were also not observed. Furthermore, OPLS-DA delivered a highly specific and sensitive model for classifying the spectra of the two examined groups. OPLS-DA results highlighted the significance of DNA and Phe-related bands for categorizing erythroid precursors and unsaturated lipids for erythrocyte-like cells (Figure S4E–G).

The haem prosthetic group production takes place in mitochondria, showing the importance of this organelle in differentiation. Considering the metabolic shift associated with erythroid differentiation and the decreasing cytochrome c signal observed in erythrocyte-like cells, we investigated the a MB probe^14,22^ mitochondrial accumulation and spectral response under single-cell Raman imaging. Cells were incubated with MB (400 nM) to assess mitochondrial localization for 15 min. Single-cell Raman imaging (532 nm) confirmed probe accumulation in mitochondria, with a distinct band at 2222 cm^–1^ corresponding to the –C≡C– vibration of the probe. However, the DOX population exhibited a higher standard deviation in the cell-silent spectral region compared to erythroid precursors (Figure 3A). The TMRE fluorescence probe, used in mitochondrial membrane potential studies, was employed to confirm these alterations. The median TMRE accumulation per cell area decreased under drug treatment (Figure 3B) and reached a similar level to that of K562 treated with the mitochondrial membrane decoupling agent FCCP for 10 min. The probe signal values show a markedly non-normal distribution, indicating distinct cell subpopulations, particularly among myeloid precursors. Increased fluorescence background in the cell-silent region (1800–2800 cm^–1^) prevented reliable probe quantification, particularly in DOX-treated cells (Figure 3A). We performed MCR-ALS image analysis to confirm mitochondrial localization, which identified strong probe accumulation within the cytochrome c-rich area. Low-intensity probe signals were also detected in lipid-rich and cytoplasmic components (Mito-ER, perinuclear, cell peripheral plasma area), consistent with the lipophilic nature of the probe. However, these signals were significantly weaker than those observed in mitochondria (Figure S6B). Other components, such as the nucleus, were not affected by the presence of the probe (Figure S6B). The percentages of high concentration of component pixels correspond to those obtained in the analysis without a probe (Figure S6A). Additionally, the proportion of high-concentration component pixels remained consistent with the prior MCR-ALS analysis without the probe, indicating that the probe’s presence did not alter overall biochemical distributions (Figure S6A).

**Figure 3:**
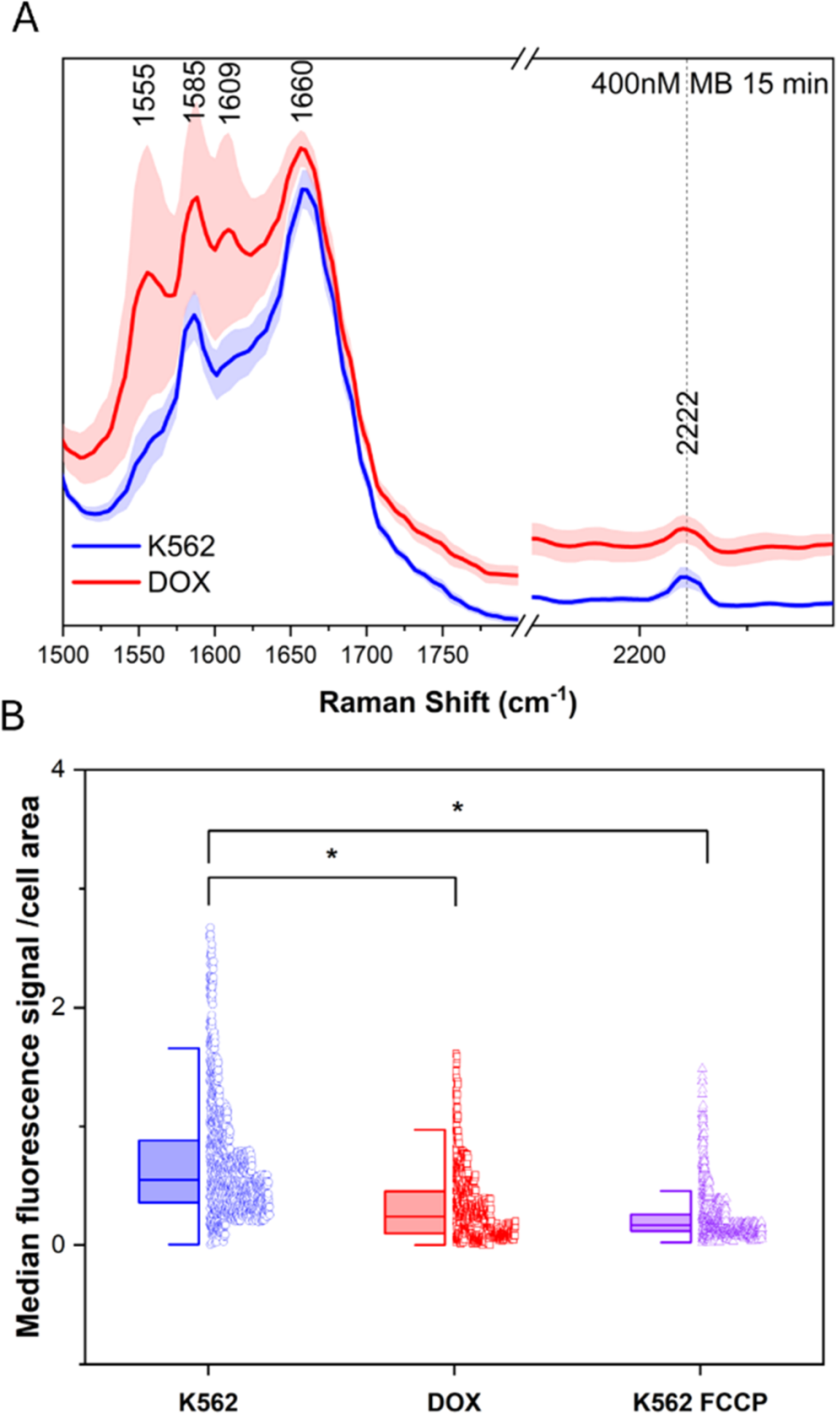
Average Raman spectra (1500–1800 and 2150–2290 cm^–1^ spectral ranges) of erythroid precursors (K562) and erythrocyte-like cells (DOX) incubated with 400 nM MB probe for 15 min (A). Median TMRE signal per cell area for 3 independent biological replicates. K562 cells pretreated with 10 μM FCCP for 10 min (K562 FCCP) were a positive control. A nonparametric median test was used to verify statistical differences at a 5% level (B).

To enhance imaging speed and analyze a larger number of cells, we transitioned from single-cell Raman imaging to hyperspectral SRS (hsSRS) microscopy. This approach enables faster spectral acquisition by collecting consecutive images at 6 cm^–1^ intervals. We processed the hsSRS data using spectral phasor analysis, successfully differentiating three distinct cellular regions: the nucleus, cytoplasm, and an intermediate region containing membrane lipids and cytoplasmic residues (Figure S8A–C). In erythrocyte-like cells, we observed circular nucleolar structures within the nucleus, which were enriched in protein and RNA signatures. This observation aligns with the increased ribosomal activity required for hemoglobin synthesis in DOX-treated cells, a process essential for erythroid differentiation.

Comparative spectral analysis (Figure S8D) revealed notable biochemical differences between cell types, particularly in the 2850–2910 cm^–1^ region, where erythrocyte-like cells exhibited higher intensity. These bands correspond to symmetric and asymmetric stretching of lipid chains, suggesting membrane remodelling or increased lipid metabolism during differentiation.

Considering MB’s potential as a differentiation probe, we examined its accumulation through hsSRS spectra within the 2150–2300 cm^–1^ range (Figure 4A). Achieving these measurements demanded a laser power of 100% and about 20 min for each acquisition, unlike the shorter exposure times needed for hsSRS imaging in high-wavenumber regions. This necessity arises due to the comparatively weaker probe signal. Interestingly, erythrocyte-like cells were more sensitive to higher laser power, displaying distorted hsSRS spectra compared to erythroid precursors. This suggests possible differences in photostability or structural properties, such as altered metabolic activity or reduced intracellular chromophores, which may affect resonance enhancement. To overcome laser power limitations, we explored single-wavenumber Raman imaging, which allows for faster acquisition without extended laser exposure, making it a practical alternative for cell classification.

**Figure 4:**
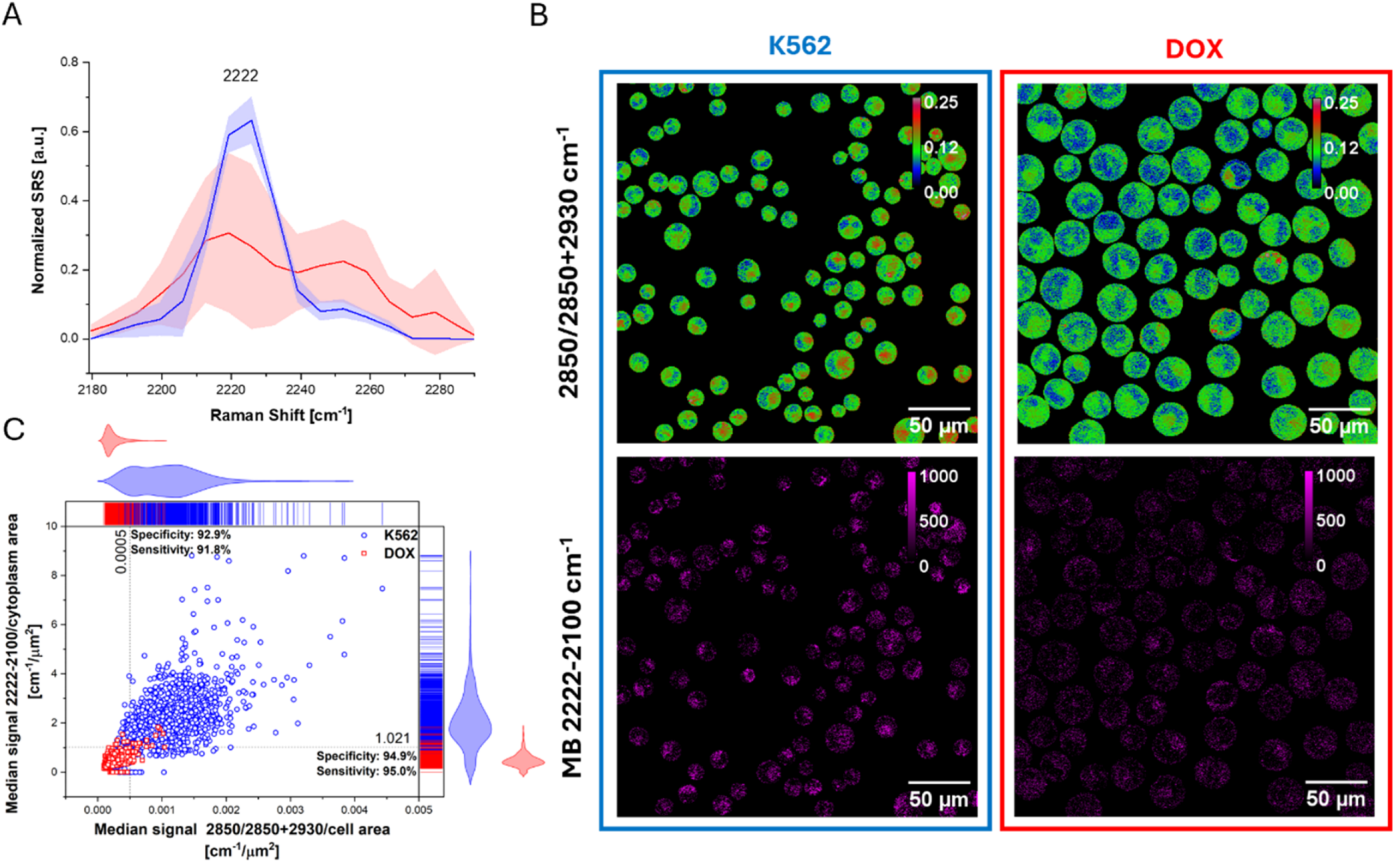
Normalized hsSRS spectra for erythroid precursors (blue) and erythrocyte-like cells (red) in the spectral range of the MB probe marker band (A) SRS images calculated for SRS intensities ratio (2850/2850 + 2930 cm^–1^), scale bar represents color-coded assignment to subcellular structures (blue-nucleus, green-cytoplasm, red-lipid rich regions) and MB distribution images. (2100 cm^–1^, the off-resonance frequency) (B). The median signal intensity normalized to total cell area 2850/2850 + 2930 cm^–1^ [cm^–1^/μm^2^] or cytoplasmic region 2222–2100 cm^–1^ [cm^–1^/μm^2^]. The dotted line indicates the thresholds obtained in the ROC analysis (C).

To develop a rapid differentiation classification method that could be useful for diagnostic support and chemotherapy effectiveness assessment, we selected two spectral markers: lipid-to-protein ratio (2850/2850 + 2930 cm^–1^), which reflects membrane remodelling and metabolic shifts during erythroid differentiation, and MB probe signal (2220–2100 cm^–1^ band) of MB scaled by the area, allowing differentiation based on probe distribution relative to cell size (Figure 4B–C). To refine classification accuracy, an off-resonance 2100 cm^–1^ wavenumber was subtracted from probe measurements to minimize background interference.

The median signal intensity, normalized to total cell area or cytoplasmic region (green in Figure 4B), provided a robust discrimination metric between erythroid precursors and erythrocyte-like cells. The median values were significantly different, as determined by the Mann–Whitney test (Figure S10). Four quadrants were generated by the classification threshold selected in the Receiver Operating Characteristic (ROC) analysis (Figure 4C). Upper right for erythroid progenitors above 1.021 (MB accumulation/area) and 0.0005 (lipid/protein ratio/area) and lower left for erythrocyte-like cells below the threshold. ROC analysis demonstrated high classification performance for both found metrics: lipid-to-protein ratio (AUC = 0.981, sensitivity = 0.928, specificity = 0.918) and MB probe/cytoplasm area (AUC = 0.991, sensitivity = 0.950, specificity = 0.949). These values indicate strong reliability in distinguishing erythroid precursors from erythrocyte-like cells, suggesting that both spectral markers could serve as diagnostic tools for monitoring erythroid differentiation and assessing chemotherapy effectiveness.

## Discussion

During the differentiation process, the cell’s metabolism and the function of its organelles may be altered to adapt to the new roles they must play in the organism. DOX-induced differentiation, as shown above, influences the nuclear structures by arresting the cell cycle, increasing the number of nucleolus, influencing the level of ROS, and initiating the synthesis of hemoglobin, which takes place in the mitochondria (prosthetic group) and the cytosol (globin part).^31^ Mitochondria, besides being responsible for producing energy, play a role in apoptosis that is altered in leukemic precursors. Therefore, we used the MB, which accumulates as a function of the mitochondrial membrane potential. The differentiation process was confirmed by increased CD235a expression, hemoglobin detection (benzidine test), and RS-based characterization of hemoglobinisation. Despite reduced cell proliferation, a high percentage of viable cells (>88%) confirmed that this concentration was noncytotoxic, supporting its role as a differentiation inducer rather than a cytotoxic agent.

While several studies have explored RS in hematopoietic cells, most have focused either on mature erythrocytes—owing to their strong hemoglobin resonance signals—or on leukemia-related phenotyping without tracking induced differentiation dynamics.^15,16^ In contrast, our study combines hsRS and SRS microscopies with a mitochondria-targeted MB to track both metabolic shifts and phenotypic changes during doxorubicin-induced differentiation. This integrative approach enables real-time, label-free monitoring of differentiation-associated biochemical events, such as hemoglobin accumulation, lipid remodelling, and mitochondrial depolarization, which are not captured by traditional flow cytometry or histochemical staining. Thus, our work addresses both biological and methodological gaps by providing a multimodal spectroscopic framework for studying erythroid maturation in live leukemia cells.

RS provided a highly sensitive and specific approach for detecting hemoglobin accumulation in erythrocyte-like cells, confirmed by resonance-enhanced hemoglobin bands. The OPLS-DA classification model effectively distinguished erythroid precursors from erythrocyte-like cells, with 0.928 sensitivity, 0.884 specificity, and a MCC of 0.808, supporting the robustness of this approach. Implemented 532 nm incident radiation is near the vibronic Q_v_ band of the visible spectrum hemoglobin, leading to RR effect and band enhancement.^6^ This allows for a lower detection limit for hemoglobin present in cells compared to the visual inspection of blue crystals in the benzidine test. Additionally, 785 nm excitation further confirmed the presence of hemoglobin, highlighting its advantage in minimizing fluorescence interference from haem and DOX while still capturing differentiation-associated spectral changes.

In contrast to the benzidine test, which detects higher hemoglobin concentrations, Raman imaging provides molecular specificity, enabling the simultaneous detection of cytochrome c, glycogen, and nuclear components. This makes Raman-based imaging a valuable complementary technique for studying erythroid differentiation. Multivariate analysis (MCR-ALS) of hsRS images revealed ten distinct biochemical components, allowing the characterization of differentiation-related metabolic shifts. Notably, the accumulation of hemoglobin exhibited a significant increase from 7%, as reported in K562, to 52% in cells treated with DOX, which is consistent with the findings of benzidine staining (9% versus 45%, Figure S1A). Furthermore, cytochrome c levels declined from 21% to 15%, indicating a metabolic transition from oxidative phosphorylation to hemoglobin biosynthesis. The glycogen-rich regions demonstrated a heightened contribution in cells treated with DOX, possibly due to metabolic adaptations occurring during differentiation. An increase in glycogen content was reported upon hemin-induced differentiation^2^ suggesting a metabolic shift. Myeloblasts are characterized by a low glycogen content when compared to the cells of healthy donors.^32,33^ Therefore, their presence in the cellular spectral profiles may indicate metabolic alterations associated with the differentiation of DOX-induced effects.

The differentiation-associated lipid remodelling was reflected in the higher contribution of choline-containing phospholipid esters and saturated lipids compared to unsaturated lipids. This may indicate changes in membrane composition, supporting the structural transformation of erythrocytes during maturation.

However, the disadvantage of MCR-ALS is a rotational ambiguity related to the possibility of multiple solutions of the bilinear decomposition. The obtained results are similar for the hsRS images of K562 and DOX compared to those of the cells incubated with MB.

The decrease in the contribution from nuclear material to the Raman from 40% in K562 cells to 21% in erythrocyte-like cells correlated with chromatin condensation and nucleation, key features of erythroid differentiation. The formation of nucleoli detected in spectral phasor analysis of hsSRS images supports increased ribosomal activity, which is required for hemoglobin protein synthesis. The nucleolus is the site where ribosomes are synthesized to produce proteins and can be correlated with the protein part of hemoglobin synthesis. We also observed differences in the SRS spectra at 2850–2910 cm^–1^, which is characteristic for C–H stretching in nucleic acids. DOX treatment also induced cell cycle arrest, likely in the G2/M phase, contributing to decreased proliferation. These changes align with previous studies showing that anthracyclines influence nuclear architecture and transcriptional regulation in differentiating cells.^3^

Respiratory capacity, mitochondrial membrane potential (Figure 4), and ROS (Figure S1E) generation change, switching from glycolytic to oxidative metabolism.^34^ Given the known role of mitochondria in haem biosynthesis,^31^ we explored mitochondrial activity using the MB Raman probe, which accumulates based on mitochondrial membrane potential. It has been demonstrated that the application of MB improves the classification sensitivity and specificity in neutrophil differentiation.^14^ MB accumulation was lower in erythrocyte-like cells (Figure 3), suggesting reduced mitochondrial activity, consistent with the transition from oxidative metabolism to hemoglobin biosynthesis. However, fluorescence from DOX and hemoglobin complicated quantification, as seen in spectral baseline distortions in the 1800–2800 cm^–1^ region. MCR-ALS analysis confirmed that the highest MB probe accumulation was observed in cytochrome c-rich areas, while lipid and cytoplasmic components had only minimal probe retention.

To minimize fluorescence artifacts, we employed hsSRS imaging, which confirmed lower probe accumulation in erythrocyte-like cells and their increased sensitivity to laser exposure. This suggests that erythrocyte-like cells undergo metabolic adaptations that influence their interaction with light-based imaging techniques. To establish a fast, reproducible method for erythroid differentiation monitoring, we developed a classification system based on SRS single wavenumber images 2850, 2930, 2220, and 2100 cm^–1^.

The first marker is related to the lipid-to-protein ratio (2850/2850 + 2930 cm^–1^), which reflects membrane composition changes. In turn, the intensity of the MB probe (2220–2100 cm^–1^), normalized to the cytoplasmic area, tracks mitochondrial activity. ROC analysis confirmed high classification performance for both spectral markers. These results suggest that this Raman-based classification model could serve as a valuable diagnostic tool for monitoring erythroid differentiation and chemotherapy response. Together, these findings not only confirm the utility of Raman-based approaches for studying erythroid differentiation but also demonstrate their potential as diagnostic tools for real-time monitoring of treatment-induced maturation in leukemia, surpassing the limitations of existing techniques.

## Conclusions

This study demonstrates that Raman-based hs imaging, SRS, and machine learning approaches can effectively characterize DOX-induced erythroid differentiation in K562 cells. By integrating biochemical profiling, spectral phasor analysis, and MB probe accumulation assessment, we provide a comprehensive understanding of the molecular changes associated with erythroid maturation. To facilitate rapid differentiation assessment, we developed a Raman-based classification model using lipid-to-protein ratio as an indicator of membrane remodelling and MB probe accumulation as a marker of mitochondrial metabolic shifts. The classification system demonstrated high accuracy, making it a promising tool for monitoring erythroid differentiation and chemotherapy response assessment.

The current diagnostic assessment of erythroid differentiation relies on time-consuming or end point-based methods, such as benzidine staining or flow cytometry for CD235a. Our Raman/SRS approach, particularly when combined with MB probe quantification, enables real-time, noninvasive detection of both structural and metabolic markers of differentiation. This positions our methodology as a promising alternative for the rapid assessment of drug efficacy in leukemia differentiation therapy models. This study underscores the potential of Raman-based spectroscopy as a noninvasive technique for monitoring erythroid differentiation. Future investigations should focus on validating this method by applying supplementary differentiation inducers, refining fluorescence compensation strategies to achieve improved quantification of Raman probes, and examining its translational applications in clinical diagnostics and the real-time monitoring of erythroid disorders.

## Supplementary Material



## Data Availability

Raw data are available at https://doi.org/10.57903/UJ/MNUAZG.

## Notes

Experimental procedures, parameters and equipment specification for all experiments presented in this article; data pertaining to the imaging and analysis (PDF)
