# Supplementary material for: Sensitive Detection and Identification Method of Erythrocyte-like Cells upon Doxorubicin Induced Differentiation with Vibrational Techniques
Source: Anal Chem. 2025 Jul 29;97(31):16966–74. doi: 10.1021/acs.analchem.5c02465 (PMC12355469; doi:10.1021/acs.analchem.5c02465)
Supplement: Supplementary file 1 [file ac5c02465_si_001.pdf]

Sensitive Detection and Identification Method of Erythrocyte-like Cells upon Doxorubicin Induced Differentiation with Vibrational Techniques

Supporting Information

Adriana Adamczyk [1,2], William Tipping [2], Olga Mazuryk [1], Duncan Graham [2], Malgorzata Baranska[1], Katarzyna Majzner [1]*,

[1] Jagiellonian University, Faculty of Chemistry, Gronostajowa 2, Krakow 30-387, Poland

[2] Department of Pure and Applied Chemistry, Technology and Innovation Centre, University of Strathclyde, 99 George Street, Glasgow, G1 1RD, U.K.

Corresponding author

* katarzyna.b.majzner@uj.edu.pl

Table of Contents

Experimental Procedures 2
Cell culturing 2
Doxorubicin induced differentiation 2
Flow cytometry 3
Benzidine Staining 3
Tetramethylrhodamine (TMRE) imaging 3
Raman Imaging 3
SRS imaging 4
Raman imaging sample preparation 5
SRS imaging sample preparation 5
Data processing 5
Erythroid differentiation assessment. 7
RS and SRS Imaging 9
Rerefences 19

Experimental Procedures

Cell culturing

K562 suspension cell line (ETACC CCL 243) was cultured in RPMI 1640 medium supplemented with GlutaMAX™ (Gibco) and 10% FBS (Gibco). The penicillin and streptomycin (Gibco) and amphotericin B (Fungizone) (Gibco) were maintained at 1% v/v. Cell suspension was maintained between 10^5-10^6 cells/mL and incubated 5% CO_2; 37°C.

Doxorubicin induced differentiation

24 h before drug treatment, 10^5 cells/ml were seeded in the six-well plate. Doxorubicin solution in full medium was added (100 nM final concentration) and cells were incubated without a change of medium for 72 h. The doxorubicin stock solution (2mM in descaled and sterile water) contributed to 0.005% water in the well.

Flow cytometry

CD235a-APC (Thermo Scientific) was used to verify the differentiation effectiveness. Additionally, the reactive oxygen species (ROS) level was established using $DCFH_2$ (Thermo Scientific). All stained cells were analysed using the standard optics of a FACSVerse™ flow cytometer and FACSuite software (Becton Dickinson, San Jose, CA). At least three independent biological repetitions of each staining were performed.

Benzidine Staining

A 0.2% TMB (tetramethyl benzidine) (Sigma) solution in 0.5% acetic acid was prepared. Before the experiment, 5 μl of 30% v/v H_2O_2 was added per 1 ml of prepared solution. Such solution was mixed 1:1 v/v with the suspension of 150,000 cells and left for 15 minutes in the dark. Cells with blue crystals were counted under a microscope, and at least 100 cells were counted.

Tetramethylrhodamine (TMRE) imaging

Samples with cells were prepared the same as for MB accumulation studies; however, they were treated for 15 min with 25 nM TMRE. After the washing step, the samples were imaged using a Confocal microscope with 561 nm excitation and detection in the 570-610 nm range. Median fluorescence values and areas were calculated using ImageJ.

Raman Imaging

Raman imaging was performed with a WITec Alpha 300 confocal Raman microscope (Oxford instruments, Abington, UK), with 1800 g/mm grating equipped with an air-cooled laser of 532 nm excitation wavelength of 50 mW power, a CCD detector (Andor Technology Ltd, Belfast, Northern Ireland), and a water dipping objective 40x (Zeiss W Plan-Apochromat, NA = 1, Oberkochen, Germany). Hyperspectral images of living cells were collected with 0.5 μm resolution and spectra integration time 0.03 s. Maximum sample study time was 45 min.

To collect Raman spectra free of haemoglobin resonance, a 785 nm excitation wavelength was used (Renishaw via Raman), with grating 1200 g/mm and 50 mW laser power. However, due to the lower energy of the excitation wavelength, which results in weaker Raman scattering, we collected spectra from several points per cell to enhance spectral quality and reduce sample investigation time. Line imaging with 1 μm step and 2 s integration time was performed in such a way to probe both the cytoplasm and nucleus region.

SRS imaging

The integrated laser system produced synchronised laser beams (80 MHz repetition rate) (picoEmerald™ S, Applied Physics & Electronics, Inc.). A fundamental Stokes beam (1031.4 nm, 2 ps pulse width) was intensity modulated by an electro-optic-modulator with >90% modulation depth, and a tunable pump beam (700–960 nm, 2 ps pulse width, <1 nm (10 cm^{-1}) spectral bandwidth) was produced by a built-in optical parametric oscillator. Spatial and temporal overlapping of the pump and Stokes beams was achieved using two dichroic mirrors and a delay stage inside the laser system and coupled into an inverted laser-scanning microscope (Leica TCS SP8, Leica Microsystems) with optimised near-IR throughput. SRS images were acquired using 40× objective (HC PL IRAPO 40×, N.A. 1.10 water immersion lens) with a 9.75–48 μs pixel dwell time over a 512 × 512 frame. The Stokes beam was modulated with a 20 MHz EoM. The forward scattered light was collected by an N.A. 1.4 condenser lens (Leica Microsystems). The SRS microscopy was performed with the Confocal Raman Scattering microscope described elsewhere[1]. Images were acquired at 12-bit image depth. 100% of laser power was used in the cell silent region with 600 V gain, and 50% was used in the high wavenumber range with 430 V on the detector and dwell time of 9.75 μs. Hs SRS images in the high wavenumber range were collected with 48 μs dwell time while in the cell silent region with 9.75 μs.

Raman imaging sample preparation

200000 cells were attached to poly-L-Lysine coated CaF_2 substrates according to protocol.[16] 3 ml of 400 nM MitoBADY in buffer was added to a dish with cells attached to substrate for 15 minutes and incubated in 37 in 5% CO_2. Substrates were washed twice and immersed in a warm buffer for Raman imaging.

SRS imaging sample preparation

200000 cells were plated onto high-precision glass coverslips (#1.5H thickness, 22 × 22 mm, Thorlabs) coated with poly-L-Lysine according to protocol[16] and if necessary 3 ml of 400 nM MitoBADY in buffer was added for 15 min. The cells were washed with fresh PBS prior to mounting them onto a glass microscope slide with a boundary of PBS. The sample was then sealed with nail polish and studied for a maximum of 40 min.

Data processing

Initial preprocessing of Raman hyperspectral images was performed in the WITec Project FIVE 5.3 (Oxford instruments, Abington, UK) and included cosmic ray removal (CRR), and Savitzki-Golay smoothing (3rd order polynomial, 7 spectral points). A background with a 3rd-order polynomial for fingerprint and high wavenumber region was applied, followed by a shape with a size of 200 in the cell silent region. Further, Raman mean cell spectra were obtained using a Python script based on the Pearson coefficient.[2] The hyperspectral image spectra were compared with the cell and background reference spectrum (Pearson coefficient 0.8) and averaged. Spectra were cut in the 400-3050 cm^{-1} range aligned and vector normalized (400-3050 cm^{-1}) in OPUS 7.0 software (Bruker Optik GmbH, Massachusetts, USA).

Discrimination models, on the other hand, were optimised in the PLS and MIA toolbox (Eigenvector Research, USA) for cell spectra with additional EMSC (mean of all spectra served as the reference spectrum, 5th polynomial to correct baseline profile) followed by the mean centring. The leave-one-replicate-out method was selected for cross-validation by removing

one entire independent biological replicate. In total spectra from 3 independent biological replicates (43 K562 and 40 DOX-treated cells) were used for model calibration and cross-validation. Permutation tests were included to ensure model significance (200 permutations). Hyperspectral Raman image analysis was also performed in the PLS and MIA toolbox. MSC median baseline correction and normalisation were used for the 400-3050 cm^{-1} range. MCR-ALS with contribution contrast constraint[3] was optimised for the 10 components. Cells from 3 independent biological replicates were pooled together into one image (43 K562 and 40 DOX-treated cells; cells incubated with MB:85 K562 and 38 DOX)

After extracting single spectra from line measurement with 785 nm excitation wavelength the spectral preprocessing on the single spectra was performed in the PLS+MIA toolbox and included despiking, Savitzki-Golay smoothing (9 points, 3rd order), automatic weighted least squares baseline (5th order) correction, normalisation (1-Norm, normalize to the sum of the absolute value of all variables), and mean centring. The model training was performed on 3 independent biological replicates and tested with a separate one. In total, spectra from 143 K562 and 128 DOX-treated cells were used for model calibration, cross-validation, and prediction. Similarly, the leave-one-out replicate method was selected for cross-validation, and the permutation test was included.

ImageJ software was used to prepare false colour assignments, scale bars and image overlays. The exact parameters of brightness and contrast were applied to compare images reliably. The SRS signal intensity per cell was determined using ImageJ analysis tool. The spectral phasor analysis was performed using a plugin for ImageJ (http://www.spechron.com/Spectral%20Phasor-Download.aspx, accessed 9th July 2025). Segmentation of the phasor plot was performed manually using regions-of-interest (ROI) to create images of discrete cellular locations. The corresponding average spectra for each ROI were vector normalized using OPUS 7.0 software.

Ratio calculation, data plotting and statistical tests were performed with Origin 2022 software.

Table S1. Summary of the acquisition parameters.

	**RS 532nm**	**RS 785nm**	**SRS**
**Laser power before the objective**	50 mW	300 mW	Pump:150 mW<br>Probe: 300mW
**Integration time**	0.03s	2s	9.75 µs-single images and HsSRS in 2100-2300 cm^{-1} range<br>48 µs- HsSRS in 2800-3050 cm^{-1}
**Spectral resolution and grating**	3 cm^{-1}, 1800g/mm,	1 cm^{-1}, 1200 g/mm	HsSRS: for 2100-2300 cm^{-1} 6 cm^{-1}, 0.4 nm, 40 images;<br>2800-3050 cm^{-1} 9 cm^{-1}, 0.6 nm, 28 images
**Objective magnification and NA**	40x, NA1	60x, NA1	40x HC PL IRAPO 40×, N.A. 1.10 water immersion lens
**Independent replicates N;**<br>**Nb of cells investigated**	3<br>43 K562, 40 DOX-treated cells	4<br>143 K562, 128 DOX-treated	HsSRS N=3<br>Classification: N=6, 1200 K562, 500 DOX-treated cells

Erythroid differentiation assessment.

After 72 h, the cell count increased for non-treated cells by 374.6%±34.0 (SE), while drug-treated cells represented 85.0%±9.4 (SE) of the initial number of cells (Figure S1 A). Based on the trypan blue exclusion after 72 h, 92.7%±2.0 (SE) of erythroid precursors and 88.2%±2.2 of K562 treated with 100 nM DOX were viable (Figure S1 B). To ensure the erythrocyte-like phenotype of DOX-treated cells, we verified the percentage of benzidine-stained cells related

to the presence of haemoglobin in the sample. The percentage of benzidine-positive cells in the DOX-treated population was 45.1%±5.4 compared to the 9.1%±1.3 (SE) in the non-treated (Figure 1C). Moreover, the expression of CD235a (glycophorin A) surface protein was elevated for a significant part of the treated population (Figure 1S B). A change in the cell size was also observed upon treatment from 163.5±1.3 to 527±6.6 μm^2 (SE) (Figure S1F). The cell area was assigned based on the CH_3 distribution to allow signal quantification per cell. The reduced cell proliferation and high percentage of viable cells indicate that this concentration is not cytotoxic. In contrast, the increased number of benzidine-positive cells and elevated levels of glycophorin A protein prove successful erythroid drug-induced differentiation. Haemoglobin presence was also confirmed by the reddish colour of the cell pellet during sample handling (Figure S2).

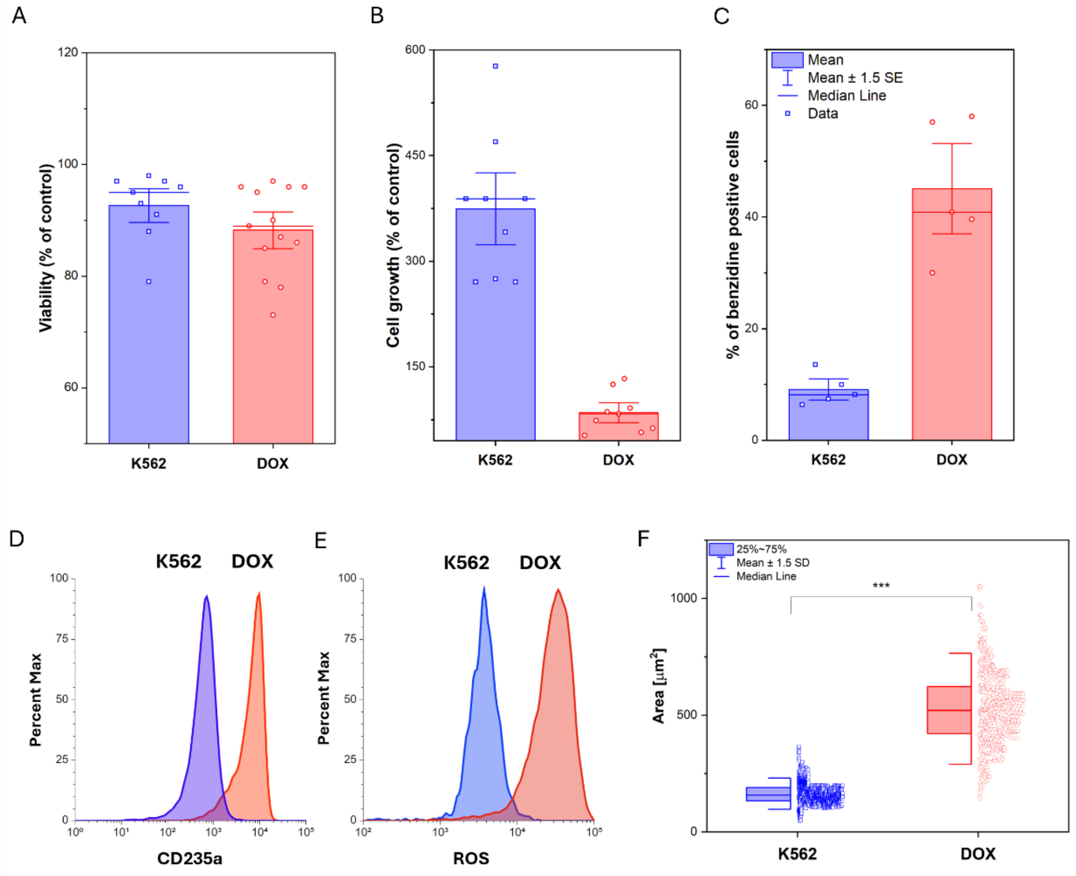

Figure S1 Cell viability (**A**), growth of erythroid precursors (K562) and 100 nM doxorubicin (DOX) treated cells after 72 h (**B**). Percentage of benzidine-stained cells (**C**). Expression of

CD235a (glycophorin A) surface marker (**D**) and ROS level after 72 h for K562 (blue histogram) and 100 nM DOX-treated cells (red histogram) (**E**). Area of the erythroid precursors (K562) and erythrocyte-like cells (DOX) (**F**). The mean difference at the 0.001 significance level was confirmed with the t-test. Values were obtained from SRS imaging.

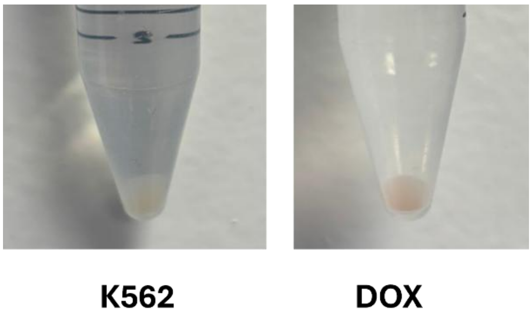

Figure S2 The colour of the cell pellet of K562 and 100 nM DOX treated cells after 72 hours.

RS and SRS Imaging

Table S2 Raman bands assignment

Band	Vibration	
**480**	Glycogen	[4,5]
**573**	Glycogen	[4,5]
**753**	v_{15}Hb, Cyt C	[6–8]
**785**	C, U, (PO_2^-) of the DNA backbone	[9]
**936**	N–Ca–C protein/glycogen	[5,8]
**1006**	δring Phe	[8]
**1040**	δring Phe	[8]
**1050**	Glycogen	[4]
**1094**	Phosphatydylocholine	[10]
**1131**	v_{22} Hb, Cyt C	[1112]
**1173-1175**	V_{30} Hb	[6,7,11,12]
**1175**	Hb	

**1180**	DNA	
**1195**		
**1260**	Amide III, proteins	[8,13]
**1290**	C	[13]
**1310**	Hb, Cyt	[8]
**1340**	v_{41} Hb	[6]
**1375**	$v_{(pyr\ half\text{-}ring)sym}$ Hb	[8]
**1400**	Hb	[6]
**1450**	δCH_2 protein and lipids	[8,13]
**1490**	Nucleic acids	[13]
**1555**	Hb	[6,7,11,12]
**1610**		[6,7,12]
**1657**	Amide I proteins	[8]
**1585**	Hb	[6,7,12]
**2910**	Glycogen	[4]

v -stretching, δ-deformation, sym-symmetric, Hb-haemoglobin, C-cytosine, U-urydine

Table S3 Thresholds used in MCR-ALS on image histograms.

**Component**	**Composition**	**Threshold**
**1**	Nuclear area	150
**2**	Lipid-rich	250
**3**	Peripheral membrane	-
**4**	Cell peripheral plasma	50
**5**	Hb-rich	50
**6**	Cyt C-rich	100
**7**	Mito-ER-rich	80
**8**	Perinuclear area	60

**9**	Glycogen-rich	100
**10**	Fluorescence bkg	30

Thresholds were selected manually to mark pixels with colour coding from the warm side of the palette (yellow, orange, red). The min-max levels for components of similar biocomposition were the same what enabled comparison between component distribution images from different experiments (with or without Raman probe MB).

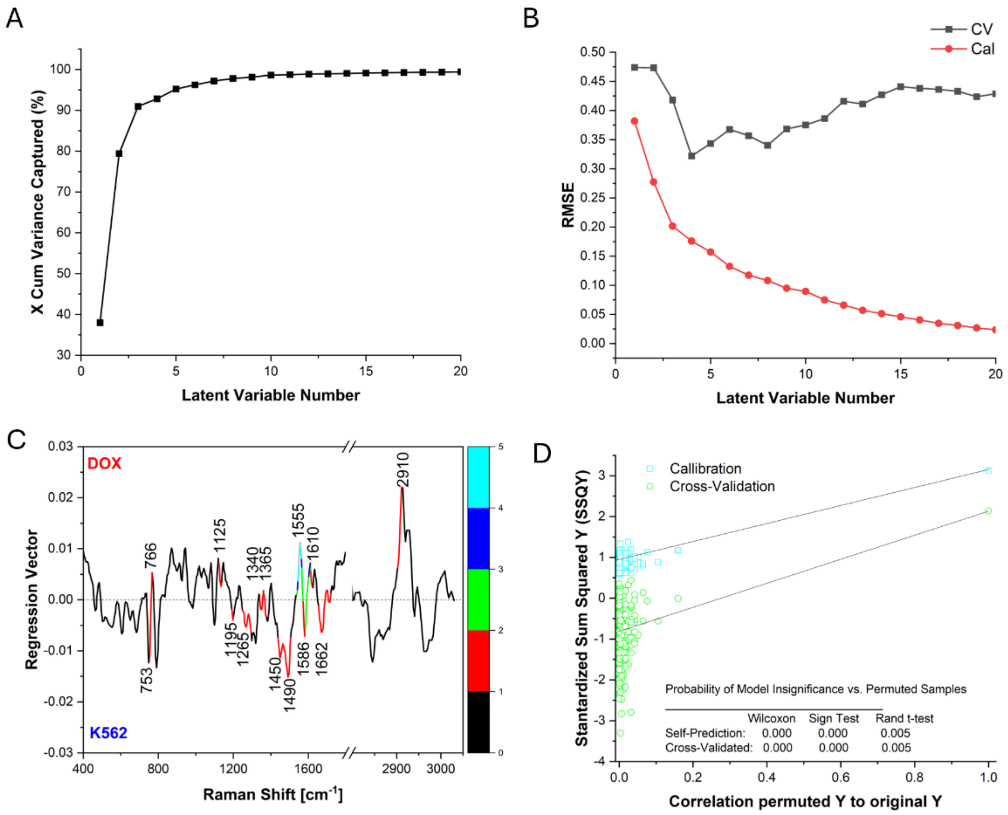

Figure S3 Orthogonal Partial Least Squares Discrimination Analysis (OPLS-DA) of erythroid precursors and erythroid-like cells shows that hemoglobin-related bands are important for classification. 4 Latent variables provided best and reliable classification. Cumulative Variance Captured for LVs (**A**) Root Mean Squared error (RSME) for Calibration (Cal) and Cross Validation (CV) (**B**). Regression vector colored by the Variance Importance in Projection values. Minima and Maxima with VIP values over 1 are important for classification (**C**) Standardized Sum Square Y ($SSQY_{,Cal/Cross\text{-}Val}$ = 1-($SSEY_{Cal/cross\text{-}Val}$ /$SSQY_{,Total}$) vs. correlation. 200 permutation were performed. (Intercept for calibration: 0, 0.831; cross-validation: 0, -0.894). Table shows probability of model insignificance. Values less than 0.05 indicate the model is significant at the 95% confidence level. The values presented confirm that it is unlikely that the original model is overfitted.(**D**)

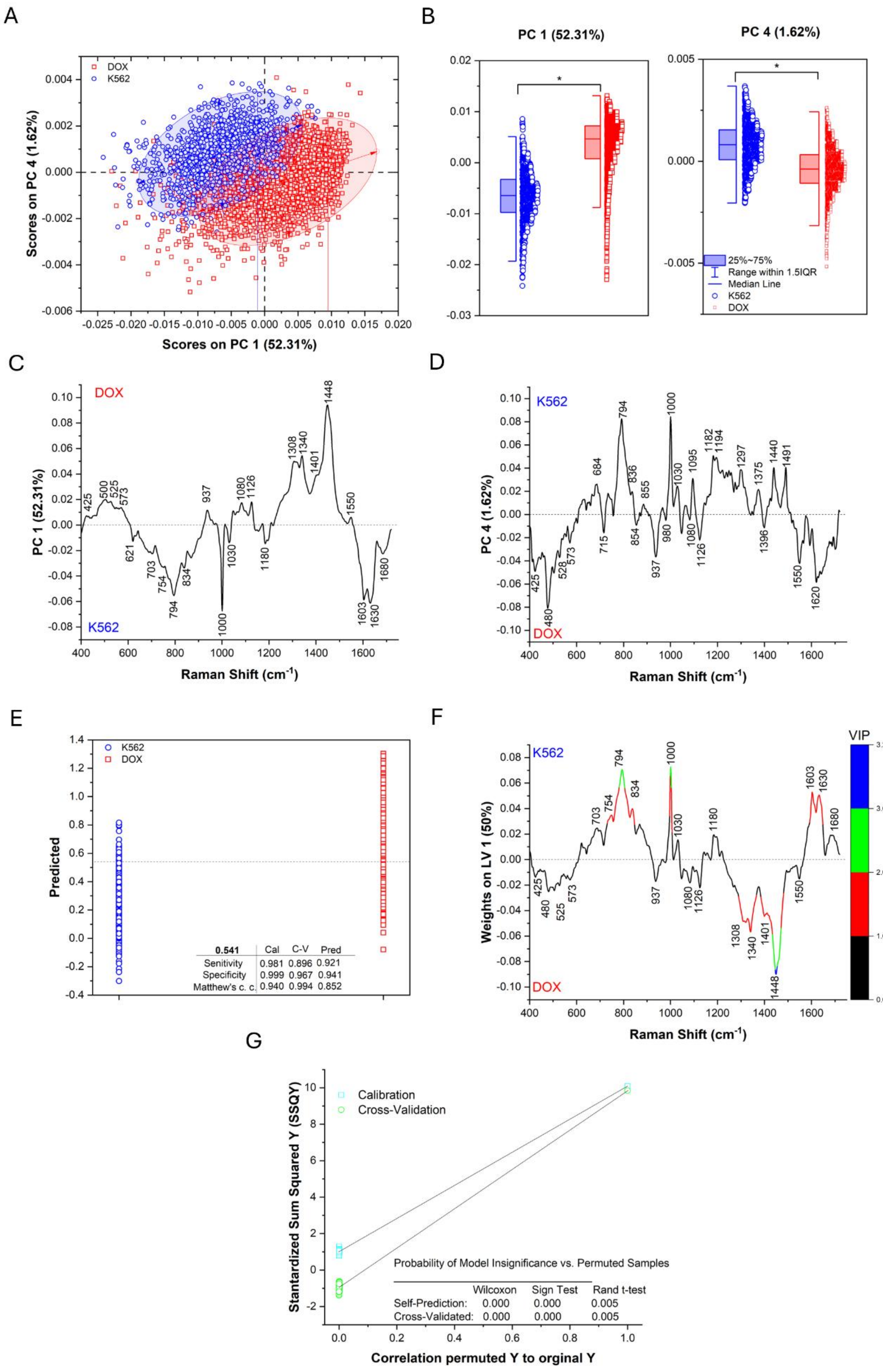
A
DOX
K562
Scores on PC 4 (1.62%)
Scores on PC 1 (52.31%)
B
PC 1 (52.31%)
PC 4 (1.62%)
*
25%~75%
Range within 1.5IQR
Median Line
K562
DOX
C
DOX
K562
PC 1 (52.31%)
Raman Shift (cm-1)
D
K562
DOX
PC 4 (1.62%)
Raman Shift (cm-1)
E
K562
DOX
Predicted
0.541 Cal C-V Pred
Senitivity 0.981 0.896 0.921
Specificity 0.999 0.967 0.941
Matthew's c. c. 0.940 0.994 0.852
F
K562
DOX
VIP
Weights on LV 1 (50%)
Raman Shift (cm-1)
G
Calibration
Cross-Validation
Standardized Sum Squared Y (SSQY)
Probability of Model Insignificance vs. Permuted Samples
Wilcoxon Sign Test Rand t-test
Self-Prediction: 0.000 0.000 0.005
Cross-Validated: 0.000 0.000 0.005
Correlation permuted Y to orginal Y

Figure S4 The erythroid precursors and erythroid-like cells were imaged with 785nm excitation to avoid the interferences related to the resonance enhancement. The points represent the point spectrum from the cell interior. Observations from 532 nm Raman imaging were confirmed. PC 1 vs. PC 4 scores plot (**A**) and box plot of each of them (**B**). Loading plots of PC 1 (**C**) and PC 4 (**D**). The significance at the 0.05 level was confirmed with the Mann-Whitney non-parametric test. OPLS-DA model and prediction (**E**). The table presents sensitivity, specificity, and Matthew's correlation coefficient for calibration (Cal), cross-validation (C-V), and prediction (Pred) with independent biological replicates at the 0.0514 classification threshold. Weights on orthogonal LV1 are coloured according to the VIP values. VIP > 1 are assigned (**F**). Standardized Sum Square Y vs. correlation. 200 permutations were performed. (Intercept for calibration: 0, 0.989; cross-validation: 0, -0.995). The table shows the probability of model insignificance. The values presented confirm that it is unlikely that the original model is overfitted. (**G**)

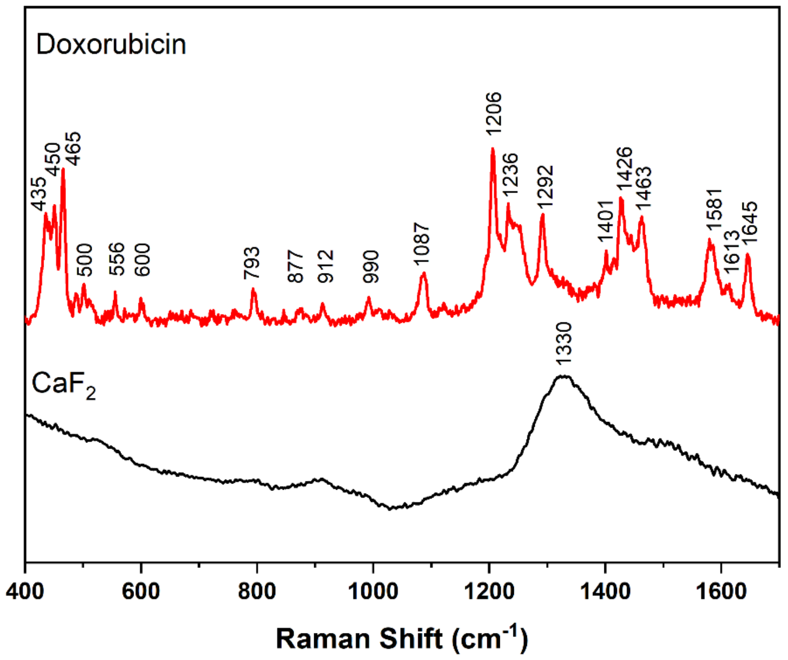

Figure S5 Average Raman spectrum of doxorubicin powder and CaF_2 from 3 different points collected with 785 nm incident radiation with 100% laser power with 40 s acquisition time.

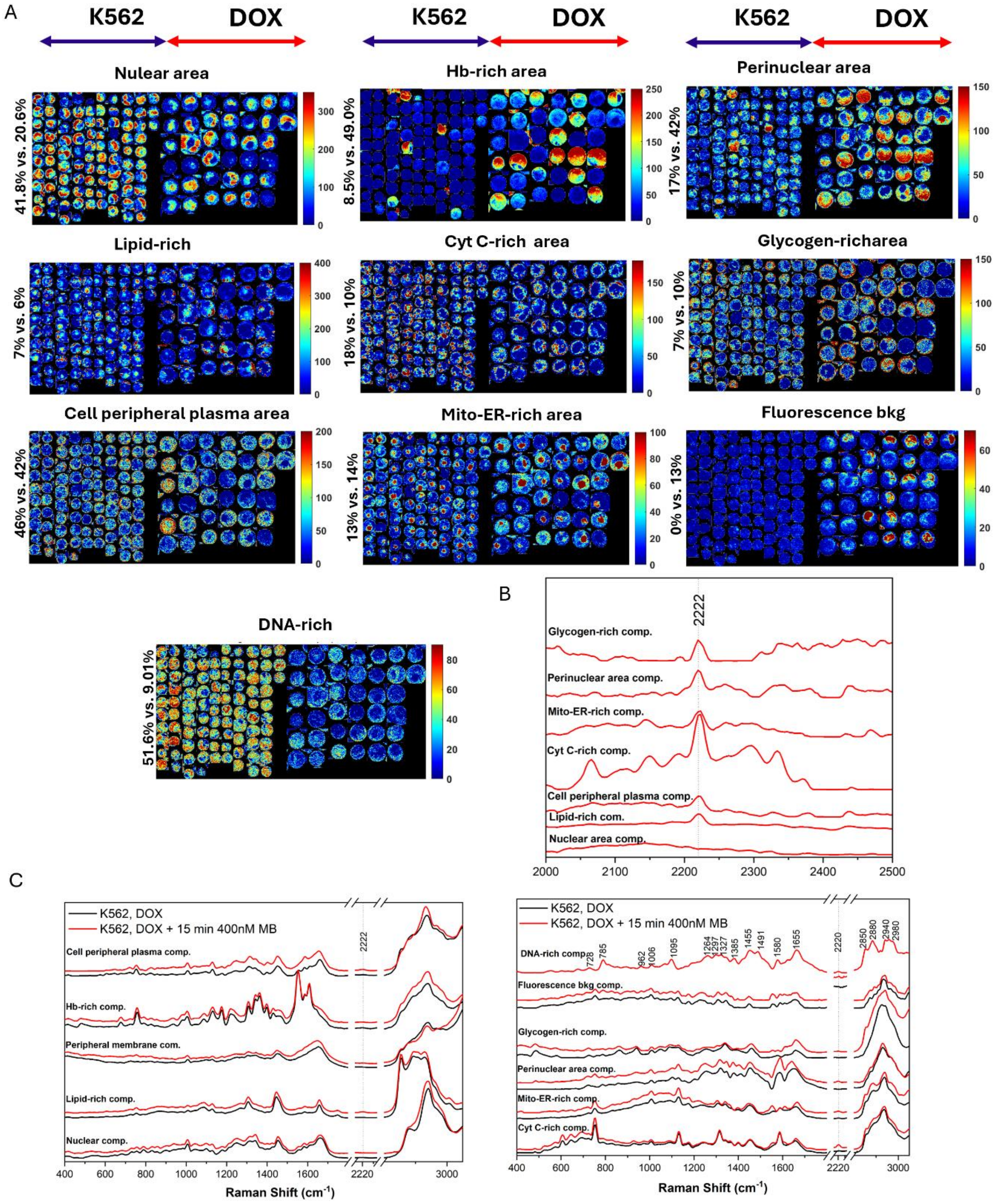

Figure S6 MCR-ALS of hyperspectral Raman images of erythroid precursors and erythrocyte-like cells treated for 15 min with 400nM of MitoBADY probe (**A**). Components distribution image and percentage of the high-intensity pixels for each population at a given threshold. Cell silent region of spectral profiles (**B**). Spectral components of cells K562 and DOX with (red line) and without (black line) MitoBADY (**C**).

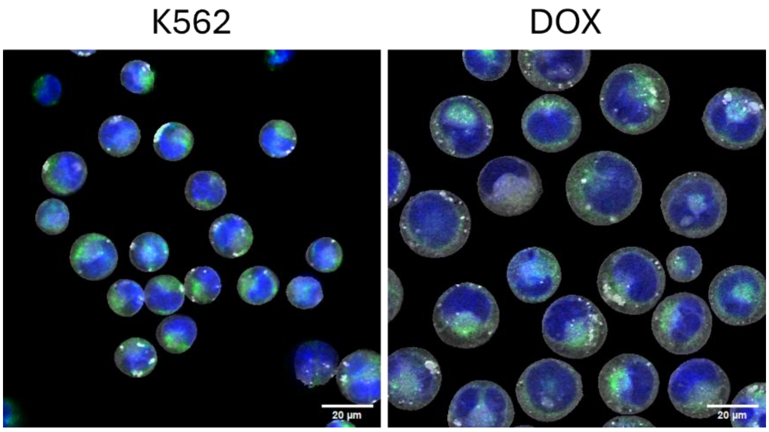

Figure S7 The overlay of the SRS signal for the 2850–2850+2930 cm^{-1} (grey), MitoTracker Orange CMTMRos (green), Hoechst33342 (blue). The cells were incubated for 15 minutes with the following mixture: 100 nM MitoTracker CMTMRos (excitation/emission: 554/576 nm) and 71.5 nm Hoechst 33342 (1:2000, stock solution: 5 mg/ml) (excitation/emission: 355/466 nm). (excitation/emission: 561/405 nm)

SRS microscopy

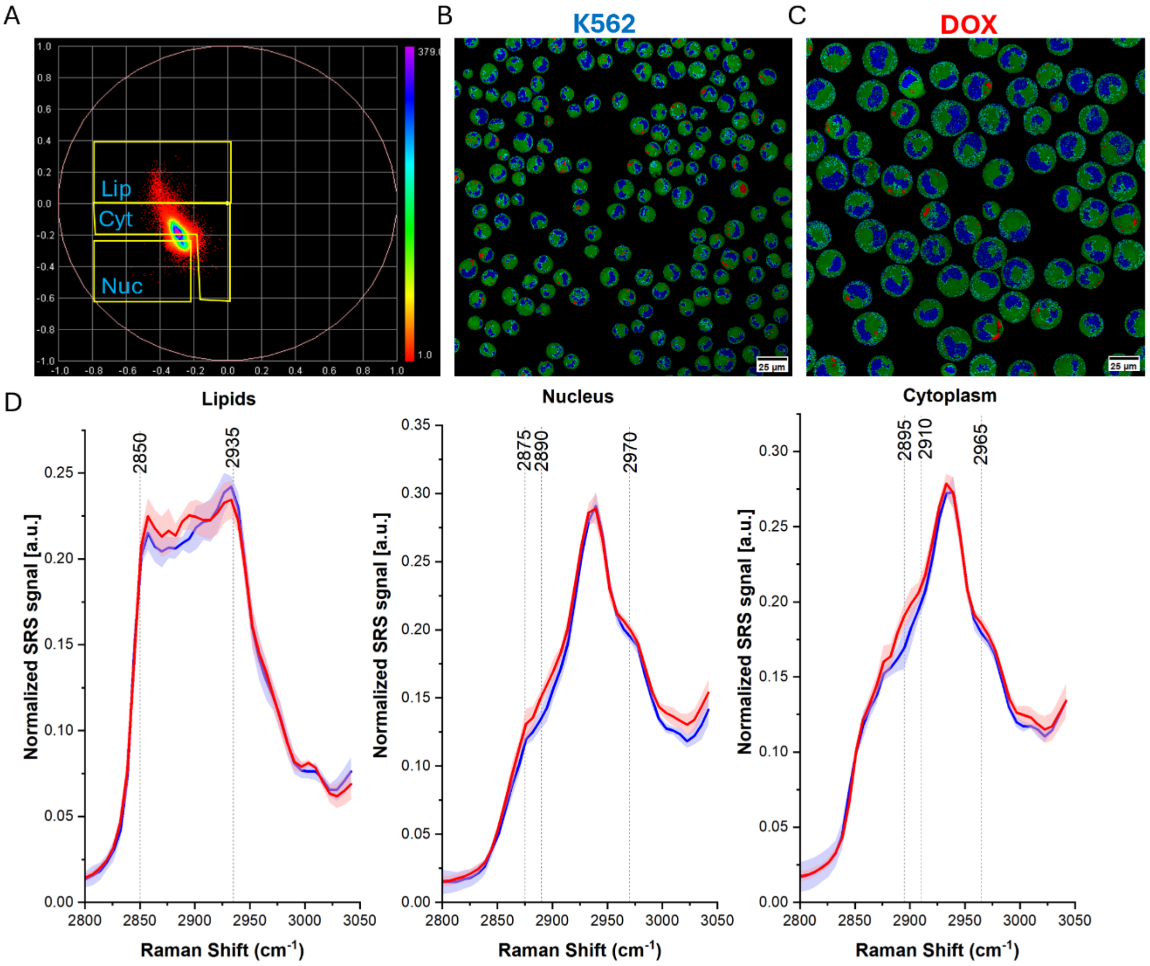

Figure S8 Phasor plot (**A**) with marked in yellow ROIs of pixels assigned to lipids (Lip), cytoplasm (Cyt), and nucleus (Nuc) used to reconstruct hsSRS images of erythroid precursors (**B**) and erythrocyte-like cells (**C**). Lipids are depicted in red, the cytoplasm in green, and the nucleus in blue. The region between the cytoplasm and nucleus is marked with cyan pixels on the phasor plot (**D**). Normalized spectra of lipids, cytoplasm, and nucleus of erythroid precursors (blue) and erythrocyte-like cells (red). SRS spectra with standard deviation reconstructed based on data from at least 3 independent replicates

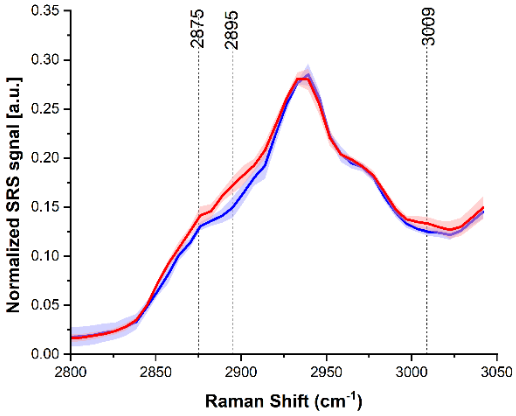

Figure S9 SRS spectrum based on phasor plot region between the pixels assigned for nucleus and cytoplasm.

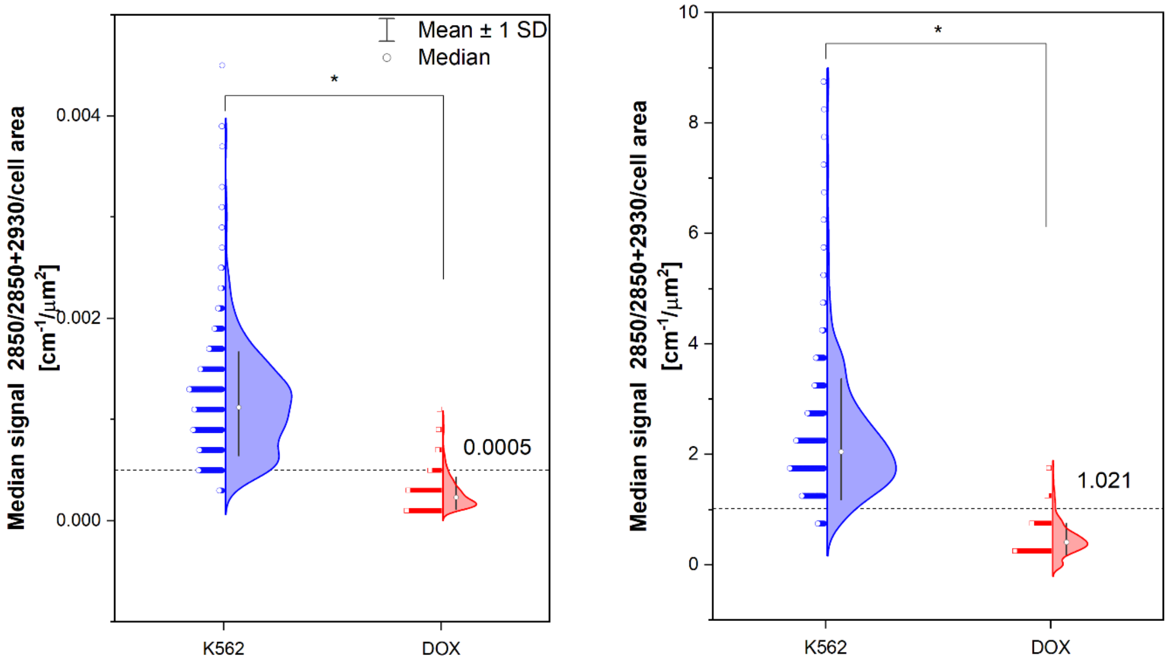

Figure S10 The violin plot for the median signal intensity, normalised to total cell area 2850/2850+2930/cell area [$cm^{-1}/\mu m^2$] or cytoplasmic region 2222-2100 cm^{-1}/cytoplasm area [$cm^{-1}/\mu m^2$]. The Kernel Smooth density distribution curve was used. The Mann-Whitney test confirmed that the means of the studied data with non-normal distribution are significantly different at the significance level of 0.05. The dotted line indicates the thresholds obtained in the ROC analysis.

Rerefences

(1) Braddick, H. J.; Tipping, W. J.; Wilson, L. T.; Jaconelli, H. S.; … Tomkinson, N. C. O. Determination of Intracellular Esterase Activity Using Ratiometric Raman Sensing and Spectral Phasor Analysis. *Anal. Chem.* **2023**, *95* (12), 5369–5376.

(2) Adamczyk, A.; Nowakowska, A. M.; Jakubowska, J.; Perez-Guaita, D.; … Baranska, M. MitoBADY-Based Raman Sensing of Neutrophil-like Cells. *Sensors Actuators B Chem.* **2025**, *422*, 136539.

(3) Windig, W.; Keenan, M. R. Angle-Constrained Alternating Least Squares. *Appl. Spectrosc.* **2011**, *65* (3), 349–357.

(4) Wiercigroch, E.; Szafraniec, E.; Czamara, K.; Pacia, M. Z.; … Malek, K. Raman and Infrared Spectroscopy of Carbohydrates: A Review. *Spectrochim. Acta Part A Mol. Biomol. Spectrosc.* **2017**, *185*, 317–335.

(5) Van Nest, S. J.; Nicholson, L. M.; Pavey, N.; Hindi, M. N.; … Lum, J. J. Raman Spectroscopy Detects Metabolic Signatures of Radiation Response and Hypoxic Fluctuations in Non-Small Cell Lung Cancer. *BMC Cancer* **2019**, *19* (1), 474.

(6) Ahlawat, S.; Kumar, N.; Uppal, A.; Kumar Gupta, P. Visible Raman Excitation Laser Induced Power and Exposure Dependent Effects in Red Blood Cells. *J. Biophotonics* **2017**, *10* (3), 415–422.

(7) Rusciano, G. Experimental Analysis of Hb Oxy–Deoxy Transition in Single Optically Stretched Red Blood Cells. *Phys. Medica* **2010**, *26* (4), 233–239.

(8) Rygula, A.; Majzner, K.; Marzec, K. M.; Kaczor, A.; … Baranska, M. Raman Spectroscopy of Proteins: A Review. *J. Raman Spectrosc.* **2013**, *44* (8), 1061–1076.

(9) Adamczyk, A.; Nowakowska, A. M.; Jakubowska, J.; Zabczynska, M.; … Baranska, M. Raman Classification of Selected Subtypes of Acute Lymphoblastic Leukemia (ALL). *Analyst* **2024**, *149* (2), 571–581.

(10) Czamara, K.; Majzner, K.; Pacia, M.; Kochan, K.; … Baranska, M. Raman Spectroscopy of Lipids: A Review. *J. Raman Spectrosc.* **2014**, *46* (1), 4–20.

(11) Dybas, J.; Chiura, T.; Marzec, K. M.; Mak, P. J. Probing Heme Active Sites of Hemoglobin in Functional Red Blood Cells Using Resonance Raman Spectroscopy. *J. Phys. Chem. B* **2021**, *125* (14), 3556–3565.

(12) De Luca, A. C.; Rusciano, G.; Ciancia, R.; Martinelli, V.; … Sasso, A. Spectroscopical and Mechanical Characterization of Normal and Thalassemic Red Blood Cells by Raman Tweezers. *Opt. Express* **2008**, *16* (11), 7943–7957.

(13) Krafft, C. Raman Spectroscopy of Proteins and Nucleic Acids: From Amino Acids and Nucleotides to Large Assemblies. In *Encyclopedia of Analytical Chemistry*; 2018; pp 1–15.
